# Screening and field performance of powder-formulated insecticides on eave tube inserts against pyrethroid resistant *Anopheles gambiae* s.l.: an investigation into ‘actives’ prior to a randomized controlled trial in Côte d’Ivoire

**DOI:** 10.1186/s12936-018-2517-9

**Published:** 2018-10-22

**Authors:** Welbeck A. Oumbouke, Innocent Z. Tia, Antoine M. G. Barreaux, Alphonsine A. Koffi, Eleanore D. Sternberg, Matthew B. Thomas, Raphael N’Guessan

**Affiliations:** 1 Department of Disease Control, London School of Hygiene and Tropical Medicine, London, UK; 2 Institut Pierre Richet (IPR)/Institut National de Santé Publique (INSP), Bouaké, Côte d’Ivoire; 3 Department of Entomology, Center for Infectious Disease Dynamics, The Pennsylvania State University, University Park, PA 16802 USA

**Keywords:** Insecticide resistance, Resistance breaking, Electrostatic coating, Powder-formulated insecticide, Residual efficacy, Eave tubes

## Abstract

**Background:**

The widespread emergence of insecticide resistance in African malaria vectors remains one of the main challenges facing control programmes. Electrostatic coating that uses polarity to bind insecticide particles is a new way of delivering insecticides to mosquitoes. Although previous tests demonstrated the resistance breaking potential of this application method, studies screening and investigating the residual efficacy of a broader range of insecticides are necessary.

**Methods:**

Eleven insecticide powder formulations belonging to six insecticide classes (pyrethroid, carbamate, organophosphate, neonicotinoid, entomopathogenic fungus and boric acid) were initially screened for residual activity over 4 weeks against pyrethroid resistant *Anopheles gambiae* sensu lato (s.l.) from the M’bé valley, central Côte d’Ivoire. Tests were performed using the eave tube assay that simulates the behavioural interaction between mosquitoes and insecticide-treated inserts. With the best performing insecticide, persistence was monitored over 12 months and the actual contact time lethal to mosquitoes was explored, using a range of transient exposure time (5 s, 30 s, 1 min up to 2 min) in the tube assays in laboratory. The mortality data were calibrated against overnight release-recapture data from enclosure around experimental huts incorporating treated inserts at the M’bé site. The natural recruitment rate of mosquitoes to the tube without insecticide treatment was assessed using fluorescent dust particles.

**Results:**

Although most insecticides assayed during the initial screening induced significant mortality (45–100%) of pyrethroid resistant *An. gambiae* during the first 2 weeks, only 10% beta-cyfluthrin retained high residual efficacy, killing 100% of *An. gambiae* during the first month and > 80% over 8 subsequent months. Transient exposure for 5 s of mosquitoes to 10% beta-cyfluthrin produced 56% mortality, with an increase to 98% when contact time was extended to 2 min (P = 0.001). In the experimental hut enclosures, mortality of *An. gambiae* with 10% beta-cyfluthrin treated inserts was 55% compared to similar rate (44%) of mosquitoes that contacted the inserts treated with fluorescent dusts. This suggests that all host-seeking female mosquitoes that contacted beta-cyfluthrin treated inserts during host-seeking were killed.

**Conclusion:**

The eave tube technology is a novel malaria control approach which combines house proofing and targeted control of anopheline mosquitoes using insecticide treated inserts. Beta-cyfluthrin showed great promise for providing prolonged control of pyrethroid resistant *An. gambiae* and has potential to be deployed year-round in areas where malaria parasites are transmitted by highly pyrethroid resistant *An. gambiae* across sub-Saharan Africa.

## Background

Wide-scale use of insecticide-based interventions such as indoor residual sprays (IRS) and long-lasting insecticide-treated nets (LLINs) has contributed to a substantial reduction in the global malaria burden in recent years [1, 2]. However, the sustainability of these approaches is now being threatened by the evolution of insecticide resistance [3, 4], creating a need for more diverse vector control tools [5].

The eave tube is a recent innovation that offers a novel approach for delivering insecticides to malaria mosquitoes [6]. The approach involves blocking the eaves of houses (if open) and inserting pieces of PVC pipe to act as ‘chimneys’ to channel the human odours mosquitoes use as cues to locate hosts for blood feeding, out of the house. When host-seeking mosquitoes enter a tube, they encounter an insert treated with an insecticide. The current version of the eave tube inserts uses electrostatic netting to hold powder formulations of insecticides. Mosquito contact with the netting results in very efficient transfer of powder particles such that even highly pyrethroid resistant mosquitoes can be killed with pyrethroid insecticides due to the overwhelming dose [7]. When eave tubes are combined with screening of windows and doors to reduce mosquito entry via other routes, the approach provides both physical protection and a killing effect, much like an insecticide treated net but at the level of the household.

Semi-field and modelling studies indicate that screening plus eave tubes (SET) could reduce transmission of malaria at community level above and beyond universal coverage of LLINs [8–10]. Based on these promising results, a cluster randomized controlled trial (CRT) is now being conducted in central Côte d’Ivoire [11] to evaluate epidemiological impact at village level. The current paper reports on a series of initial studies to screen a range of candidate insecticides for use in this trial, together with an evaluation of potential residual activity of a smaller number of promising insecticides to select a final product and inform likely retreatment frequency for the CRT.

## Methods

### Mosquitoes and insecticides

Experiments were performed with *Anopheles gambiae* mosquitoes collected from a rice growing area adjacent to the M’bé experimental hut station in central Côte d’Ivoire, approximately 40 km north of the city of Bouaké. These rice fields provide mosquito-breeding habitat year-round. A comprehensive characterization of the local mosquito population showed that the M variant of the *An. gambiae* complex, now referred to as *Anopheles coluzzii*, is predominant in the area and exhibits high levels of resistance to pyrethroid and carbamate insecticides [12, 13]. Recently, over 1700 fold resistance against deltamethrin was detected in the M’bé population of *An. gambiae* compared to the Kisumu laboratory strain, using adapted CDC bottle assays [14]. The high resistance intensity exhibited by this vector population makes it a good strain for testing potential resistance breaking chemistry or novel insecticide delivery systems, such as the electrostatic coating technology. In the experiments described below, mosquitoes were collected as larvae and pupae from breeding sites around M’bé and reared to adult in the insectary of the Institut Pierre Richet (IPR) in Bouaké, under ambient climatic conditions. Five-day-old sugar-fed only female mosquitoes were used in all laboratory and semi-field assays.

The list of insecticides initially screened for residual performance is given in Table 1. Overall, 11 products belonging to six insecticide classes (pyrethroid, carbamate, organophosphate, neonicotinoid, entomopathogenic fungus and boric acid) were tested. The products were selected for testing based on, commercial availability as pest control products, however a handful of experimental formulations were also tested. All the insecticides evaluated were powder formulations.

**Table 1:** List of insecticides initially screened for residual performance against pyrethroid resistant *Anopheles gambiae* M'bé strain

Commercial name (supplier)	Active ingredients (dose)	Chemical classes
Actellic (Syngenta, Switzerland)	Pyrimiphos methyl (1.6%); thiamethoxam (0.36%)	Organophosphate; neonicotinoid
NA	Azamethiphos (10%)	Organophosphate
NA	*Beauveria bassiana* (10%)	Fungus
Ficam D (Bayer, Germany)	Bendiocarb (1.25%)	Carbamate
BISTAR 10 WP (FMC India)	Bifenthrin (10%)	Pyrethroid
BorActin (Rockwell labs Ltd, USA)	Orthoboric acid (99%)	Boric acid
Tempo Ultra (Bayer, Germany)	Beta-cyfluthrin WP (10%)	Pyrethroid
Spritex (Denka International BV, Barneveld, The Netherlands)	Deltamethrin (0.25%)	Pyrethroid
Drione (Bayer, Germany)	Pyrethrin (1%); Piperonyl Butoxide (10%)	Pyrethroid; synergist
NA	Permethrin (25%)	Pyrethroid
Sevin (TechPac LLC, Atlanta)	Carbaryl (5%)	Carbamate

Commercial names are provided for insecticides that are available on the market; NA indicates that the insecticide was an experimental formulation and not a commercially available product

### Application of insecticide powders on eave tube inserts

Eave tube inserts that fit into locally produced PVC tubes have been designed with electrostatic netting attached to a polyethylene frame consisting of a plastic circle with six spokes and a central protruding node (see [9] for images of the insert design). The frame provides physical support to the netting and allows easy insertion inside eave tubes. This prototype was used in the present study to investigate the persistence of insecticide applied on eave tube insert.

Candidate active ingredients were applied on eave tube inserts manually; 5 g of each ‘*active*’ (powder-formulated insecticide) was weighed and poured evenly onto an eave tube insert placed in the middle of a 20 cm long PVC tube. To prevent active from falling through the tube, both ends of the pipe was sealed off with a plastic lid and the tube was then shaken by hand for 1 min. To allow for adequate distribution of the insecticide on the two sides of the insert, the tube was turned every 10 s. The tube was then put on a table for 2 min to allow the dust to settle and adhere to the insert, and then the treated insert was moved to a clean tube and shaken for 15 s to remove any excess of powder. After treatment, the insert was placed in a third, clean tube. Four to six inserts were treated for each insecticide; approximately 4 g of powder were collected after treatment, leaving approximately 1 g of powder on the insert. An excess of powder was used during treatment to ensure thorough saturation of the inserts with the powders. Inserts were tested 1 day post-treatment (T0), then kept for subsequent monitoring of residual efficacy at regular intervals. To better approximate decay rates under realistic conditions, the inserts were kept individually in eave tubes inserted in holes drilled at eave level in an experimental house on the IPR campus. The inserts were stored in these tubes throughout the testing period and removed only for persistence monitoring.

### The “eave tube” bioassay

**Fig. 1:**
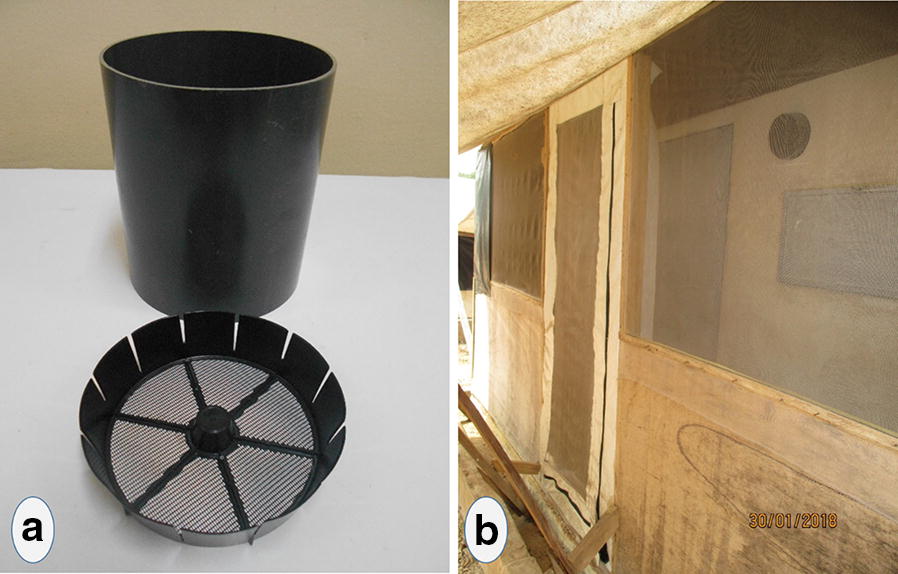
**a** Photo of the components of the eave tube assay; **b** Picture of the experimental hut fitted with eave tubes

This bioassay method uses a 20 cm long piece of PVC tube with an insecticide-treated insert placed in the tube such that it is flush with one end of the pipe (Fig. 1a). The opposite end of the tube is fitted with untreated netting to keep mosquitoes inside of the tube, and mosquitoes are introduced into the tube on this clean end using mouth aspirators. A host cue is placed behind the treated insert and the mosquitoes are allowed to recruit freely to the insert over a fixed period of time. This experimental set up was designed to simulate the interaction between mosquitoes and eave tube inserts in the field, where heat and odour cues draw host-seeking female mosquitoes into the tube where they then make contact with the insecticide-laden insert (see [15] for a similar methodology).

### Initial screening of powder insecticides

The aim of this set of experiments was to identify chemicals that retained efficacy against pyrethroid resistant mosquitoes for at least 4 weeks post-treatment. Persistence assays were performed on a fortnightly basis, and insecticides with significant decline in residual activity over the testing period were dropped from further testing. A total of ~ 60 unfed female mosquitoes aged 4–5 days were exposed in batch of 15 to each insert for 3 min using the eave tube bioassay. A hand was used as the attractive cue behind the treated insert. To eliminate any potential biases from differential attractiveness of volunteers, hand from the same individual was used in all assays. Exposure to an untreated insert served as the control. At the end of the exposure period, mosquitoes were released in netted cages with access to a 10% sugar solution on cotton pads. Mortality was scored after a 24 h holding period, except for the fungus-exposed group, which was scored 7 days later.

### Persistence monitoring

The only insecticide that persisted for 1 month during the initial screening was 10% beta-cyfluthrin. New inserts were treated with 10% beta-cyfluthrin and residual activity was monitored at approximately monthly intervals for 12 months using the same eave tube bioassays, but with some refinement of the protocol. The three modifications were: (1) the host cue was changed from a hand to a bottle filled up with boiling water and wrapped in a worn sock (worn over night), to allow for more assays to be run in parallel, (2) female mosquitoes were deprived of sugar 6 h prior to the bioassay to maximize host-seeking behaviour, and (3) the duration of the bioassay was extended from 3 min to 1 h. Although mosquitoes remained inside the tube for 1 h, it is important to note that the actual contact time was still determined by the host-seeking response of each individual mosquito. Approximately 60 mosquitoes (four replicates of 15 mosquitoes per tube) were tested. At the end of the 1 h behavioural assay, mosquitoes were transferred to observation cages, supplied with 10% sugar water solution, and mortality scored 24 h.

### Supplementary experiments

Results from residual efficacy assays show that 10% beta-cyfluthrin was the longest lasting chemical when applied on eave tube inserts. To further explore the vector control potential of this insecticide formulation, additional experiments were performed in a semi-field setting and in the laboratory using reduced contact times.

#### Field performance of insecticide-treated insert

Experiments were conducted at the M’bé phase II experimental hut station between June and September 2017 using experimental huts constructed to the West African design [16]. The huts are 3.25 m long, 1.76 m wide and 2 m high. The interior walls of the huts are made of concrete brick, with a corrugated iron roof. A plastic cover was affixed onto the roofing as ceiling. Each hut was built on a concrete base with a water-filled moat, to protect against invertebrate predators. The huts were customized to allow evaluation of eave tube inserts; namely, six holes were drilled at eave level (1.7 m from the ground) on three sides of the hut (two holes on each side). Eave tubes were fitted into the holes and inserts freshly treated with 10% beta-cyfluthrin were placed in the tubes. To allow for the recapture of mosquitoes after contact with the eave tube inserts, the huts had to be in an enclosed structure (Fig. 1b). A wooden frame was erected on the concrete base, 50 cm from the exterior wall of the hut. Plastic sheeting was used as a roof on the enclosure, and extended beyond the edge of the enclosure as an awning, to protect against rain entering the enclosure. The bottom half of the frame was made out of wooden panels and the top half was screened with polyethylene netting. White plastic sheeting was installed on the floor of the enclosure to facilitate the collection of dead mosquitoes. The door of the enclosure was positioned on the front side of the hut and closed with a zipper to prevent mosquitoes escaping.

Overnight release-recapture experiments were conducted in two modified experimental huts, situated 50 m apart. In the first experiment, six inserts treated with beta-cyfluthrin were installed in one experimental hut and six untreated inserts were placed in tubes in the second experimental house. Two adult volunteers were recruited from nearby villages to sleep in the huts. During the experiment, sleepers were rotated between the two huts. Before the start of the experiment, study participants slept in the experimental huts for a week to build up human odours and maximize mosquito host-seeking response. At 20:00, volunteers entered the huts to sleep under intact, untreated net. A total of 100, 5 day-old female *An. gambiae* (M’bé strain) were released into each enclosure 15 min after volunteers retired to their respective huts. Mosquitoes were sugar-starved for 6 h prior to the release, but still provided tap water to prevent desiccation. In the following morning, at 05:00, mosquitoes were recaptured both inside the experimental huts and within the enclosures using flashlights and aspirators. Live recaptured mosquitoes were subsequently held in netted plastic cups and supplied with 10% sugar solution. Survival was monitored for 24 h.

### Measurement of mosquito host-seeking response in the enclosure

To assess how many mosquitoes actually enter the eave tubes and came into contact with the inserts over the course of a night, a second experiment was conducted using fluorescent powder. The procedure for the experiment was similar to that described above, except that the inserts were treated with a non-toxic fluorescent dust instead of beta-cyfluthrin. The procedure for applying the fluorescent dust was similar to that used for hand-treating insert with powder insecticide as described in an earlier section. Again, the experimental huts were fitted with 6 eave tube inserts and 100 sugar-starved *An. gambiae* M’bé mosquitoes were released in each enclosure each study night. To prevent cross-contamination with the fluorescent powder, mosquitoes were caught individually using clean haemolysis tubes. Recaptured mosquitoes were killed with chloroform and their bodies subsequently checked for fluorescent particles, indicative of contact with treated inserts, using a UV light microscope (Dino Lite Premier, USA). A third experiment was also conducted where eave tubes were simply left open overnight to estimate how many mosquitoes passed through the tubes. The following morning at 05:00, the volunteers blocked the eave tubes using untreated inserts and mosquitoes inside and outside the hut were collected and counted.

### Short contact assays

Unlike house walls, where a mosquito might rest for a longer period of time, the time that vectors spend in contact with an eave tube insert could be relatively transient [17, 18]. Overnight survival in the enclosures with insecticide-treated inserts could indicate either that the mosquito did not come into contact with a treated insert or that it did not stay in contact long enough to pick up a lethal dose.

Likewise, while the presence of coloured particles on a recaptured mosquito does indicate contact with the eave tube insert, the absence of fluorescent particles could indicate either no contact, or that the mosquito did not stay in contact long enough to be contaminated with a visible amount of particles.

To evaluate whether beta-cyfluthrin can kill even with brief contact, individual mosquitoes were exposed to freshly treated inserts using the same modified eave tube bioassay. A range of exposure time (5 s, 30 s, 1 min and 2 min) was tested on 6 h sugar-starved 5-day-old female *An. gambiae* M’bé. A transparent tube was used instead of a standard PVC tube, to enable direct observation of mosquito behaviour within the tube and to allow measurement of contact duration using a stopwatch. A total of 52 mosquitoes was tested individually for each time period. Following exposure, mosquitoes were removed from the eave tube and housed in 150 mL plastic cups and provided with sugar solution. Mortality was scored 24 h post-exposure.

To test whether a contact time of only 5 s is sufficient for fluorescent particles to transfer from the insert to the mosquito, 50 female *An. gambiae* mosquitoes were exposed individually to inserts treated with fluorescent powder using the same modified eave tube assay. After 5 s of contact, the mosquito was removed and the body examined under UV light for the presence of coloured particles.

### Statistical analysis

Data were entered into an excel spreadsheet and transferred into the R statistical software version 3.4.0 for analysis. The decline in efficacy over time across insecticides was analysed using Bayesian generalized linear models (BGLMs) with the “arm” package. Insecticide treatments were included in the model as explanatory variable and mosquito mortality as the outcome. Interactions between insecticides and persistence testing intervals (time since treatment) were also included in the models. Pairwise comparisons were performed with the final model using the “multcomp” package in R. For the release-recapture experiments, generalized linear mixed models (GLMMs) with a binomial distribution and a logit link function was fitted to the data using the “lme4” package for R. Treatment and enclosure were included as fixed effects and sleepers were included as a random effect. Data from the short contact eave tube assays were analysed using Bayesian generalized linear models with a binomial distribution.

## Results

### Initial screening of powder insecticides

**Fig. 2:**
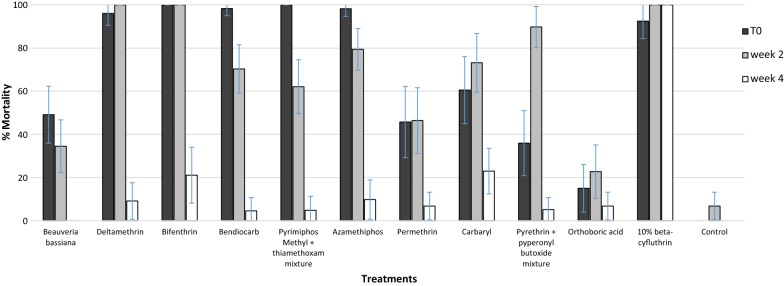
Weekly mortality rates of pyrethroid resistant *Anopheles gambiae* M’bé strain after exposure to insecticide treated insert using 3 min eave tube assay. Error bars indicate the confidence intervals for the different proportions on the graphs

Figure 2 shows the results of the eave tube bioassay tests with the 11 initial candidate powder insecticides, tested at T0, 2 weeks and 1 month post-treatment against the pyrethroid resistant *An. gambiae* M’bé strain. Comparing the 11 insecticides at T0 and 2 weeks post-treatment, most killed a significant proportion (45–100%) of *An. gambiae* mosquitoes. However, there was a significant (P < 0.05) decline in activity 4 weeks after treatment, with mortality dropping below 25% for almost all of the insecticides. In contrast, beta-cyfluthrin retained full residual activity (100% mortality) over the screening period of 1 month.

### Persistence monitoring

**Fig. 3:**
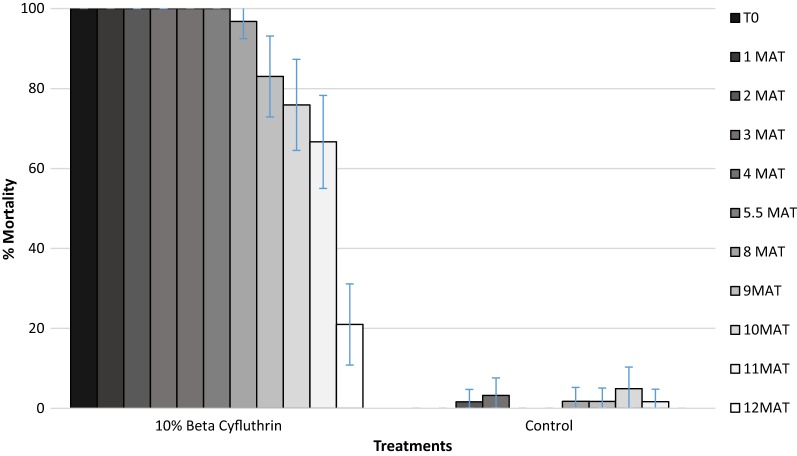
Residual activity over 12 months of 10% beta-cyfluthrin (selected from initial screening) on insert against pyrethroid resistant *Anopheles gambiae* from M’bé. Error bars indicate the confidence intervals for the different proportions on the graphs (*MAT* months after treatment)

Based on the initial screening, beta-cyfluthrin was selected for its persistence on inserts over 12 months; the results are summarized in Fig. 3. Beta-cyfluthrin was highly effective, continuing to kill > 80% of *An. gambiae* up to 9 months post-treatment. Mortality of *An. gambiae* declined steadily over time down to 67% by month 11 and 20% by month 12.

### Experimental hut evaluations

The proportions of *An. gambiae* mosquitoes recaptured in the experimental hut enclosures are presented in Table 2, both for the experiment using insecticide-treated inserts and for the one using inserts treated with fluorescent dust. Table 2 also presents the proportions of mosquitoes found dead (insecticide treatment) or recaptured with fluorescent dust particles.

**Table 2:** Release-recapture of pyrethroid resistant *An. gambiae* within enclosure at M’bé, Côte d’Ivoire

Treatment	Total released	% recaptured (95% CI)	% immediate mortality (95% CI)	% 24 h mortality (95% CI)	% with fluorescent dust (95% CI)
Untreated insert	395	90.38 [87.5–93.3]	1.12^a^ [0.03–2.21]	2.8^a^ [1.1–4.5]	–
10% beta-cyfluthrin treated insert	389	84.31 [80.7–87.9]	55^b^ [49.6–60.4]	64^b^ [58.8–69.2]	–
Fluorescent dust-treated insert	790	87.6 [85.5–89.7]	–	–	44.4 [40.7 – 48.1]

* Values in the same column not sharing a letter superscript differ significantly (P < 0.05, GLMMs)

Mosquito recapture rate was consistently high in all experiments (more than 80%). It is possible that a few mosquitoes escaped through the door of the enclosure during release, thus accounting for the small difference in number between mosquitoes released and that recaptured.

Mortality with the untreated control inserts was < 5%. When inserts treated with beta-cyfluthrin were used, about half of the mosquitoes tested died by the morning of collection (55% immediate mortality) and this increased to 64% by 24 h post-exposure, but the difference between immediate mortality and 24 h mortality was not significant (P > 0.05).

Results from the experiment using the fluorescent powder showed that, on average 44% of mosquitoes released in the enclosure had coloured particles on their body after recapture. This suggests that slightly less than half of the released mosquitoes made contact with the inserts overnight. Given that this is similar to the mortality observed when beta-cyfluthrin was used in the experimental huts (44% with coloured particles versus 55% immediate mortality with beta-cyfluthrin), this suggests that all of the mosquitoes encountering the insecticide-treated inserts were killed. When eave tubes were left open, > 75% of mosquitoes were caught inside the experimental hut. This indicates that, in the absence of the inserts, the majority of mosquitoes will pass through the tubes overnight.

### Short contact assay

**Fig. 4:**
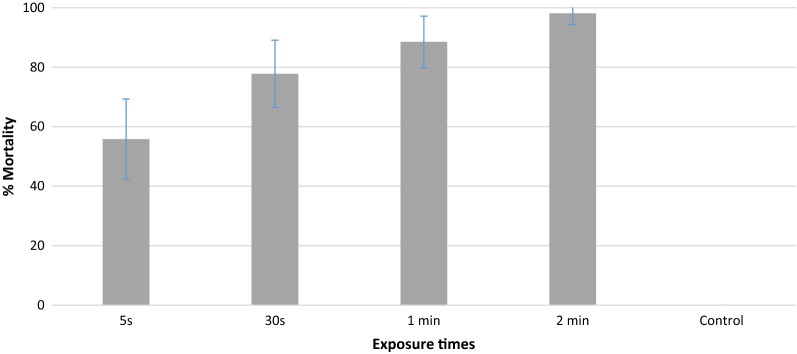
Exposure time and induced mortality of individual pyrethroid resistant *Anopheles gambiae* from M’bé with 10% beta-cyfluthrin treated insert. Error bars indicate the confidence intervals for the different proportions on the graphs

Figure 4 shows the 24 h mortality of *An. gambiae* mosquitoes after 5 s, 30 s, 1 min or 2 min exposure to inserts freshly treated with beta-cyfluthrin. There was a positive relationship between exposure duration and mortality, i.e. the longer the exposure time the higher the mortality rate. Percent mortality was 56% with the shortest exposure time (5 s), and increased significantly to 88.5% when contact time was increased to 1 min (P = 0.003). A 2-min contact with a freshly treated insert was sufficient to produce almost 100% mortality in a pyrethroid resistant *An. gambiae* strain, but the difference in mortality between 1 min and 2 min exposure was not significant (P > 0.05). There was no mortality in the control group. When mosquitoes were exposed for just 5 s on inserts treated with fluorescent dust, 100% of mosquitoes were contaminated with the coloured particles.

## Discussion

Malaria elimination will require innovative vector control tools that are not compromised by insecticide resistance. The eave tube is part of a new mosquito control strategy that involves screening windows, closing eaves, and the targeted delivery of insecticide on eave tube inserts. The intervention will be trialed in Côte d’Ivoire to test whether it can impact malaria incidence. The study presented here was designed, in part, to identify a suitable insecticide for use in the trial, and to explore a diversity of insecticides that could potentially be used in the eave tubes for prolonged control of insecticide resistant anopheline mosquito populations.

Results from residual efficacy bioassays show that the majority of insecticides tested in the present study produced significant mortality (45–100%) in the local M’bé strain of *An. gambiae* mosquitoes, when freshly applied on eave tube insert. This confirms that a wide range of actives from diverse insecticide classes could be successfully applied on electrostatic netting for effective control of insecticide resistant malaria vectors and provides further evidence of the resistance breaking potential of the technology [7].

While most candidate actives were highly effective at killing mosquitoes immediately following treatment, only one (10% beta-cyfluthrin) retained efficacy beyond 1 month. Previous studies with some of the same insecticides have reported longer residual activity than what was observed in the present study but this could be due to the difference in the nature of the substrate (electrostatic netting versus walls). The rapid loss in efficacy observed with some actives could also be due to factors that are known to degrade insecticides used during indoor residual spraying campaign, including temperature, humidity and UV-light [19]. The underlying mechanism for the rapid decay that was observed with some actives should be evaluated in further studies. However, different formulations could help mitigate some of these factors. For example, the use of UV protection additive could prevent insecticide breakdown due to photolysis and prolong the effective lifespan of chemicals. Although candidate actives were exposed to environmental conditions similar to those in local villages, persistence could still differ for a number of reasons when the insecticides are deployed in the field. For example, exposure to smoke from cooking in real houses could impact the long-term insecticidal efficacy of chemicals deployed in the eave tube. This issue has also been reported with insecticide-treated durable wall lining, where the efficacy can be undermined by dirt accumulation [20]. This emphasizes the need for continued monitoring of persistence and timely re-treatment of inserts once efficacy starts to decline.

Although the focus of this study was on readily available formulations of insecticides, there is clearly an opportunity for reformulating or repurposing a number of active ingredients for use in eave tubes. This could be useful, for example, in resistance mitigation and management where one of the recommended strategy is the use of unrelated insecticidal compounds in rotations or mosaics to delay the spread of insecticide resistant genes [21, 22]. Additionally, a diversity of active ingredients suited for deployment in eave tubes could be useful for addressing constraints on IRS. The relatively high cost of non-pyrethroid insecticide formulations coupled with a proposed reduction in IRS funding will result in much fewer houses being sprayed across sub-Saharan Africa [23], but only a small amount of insecticide is needed to protect a house with eave tubes. Moreover, most insecticides are short-lived when applied on mud wall, which is common in most rural endemic areas across sub-Saharan Africa. This may be less of a problem with the eave tube technology given that insecticides are deployed on substrate with standard characteristics.

In the experimental huts, beta-cyfluthrin produced 55% mortality of pyrethroid resistant *An. gambiae* mosquitoes. Although the mortality observed in the experimental huts is consistent with findings from previous studies [8, 9], mortality was much higher in laboratory bioassays. This could be either due to a percentage of mosquitoes not entering the tubes over the course of the night or that contact with the treated inserts was too transient for the mosquito to pick up a lethal dose of insecticide. When inserts were treated with fluorescent powder and placed in the experimental huts, the proportion of mosquitoes that contacted the fluorescent dust (44%) was similar to the mortality (55%) induced by beta-cyfluthrin treated inserts. This suggests that not all female mosquitoes came into contact with the treated inserts but those females that contacted the tube died, and this would have happened within the first 2 min of exposure. In other words, overnight mortality is likely determined by the probability a mosquito will come into contact with the treated insert rather than the probability the mosquito will die given it has contacted a treated insert (if the inserts are freshly treated with insecticides). Interestingly, the proportion of mosquitoes entering through open tubes (> 75%) was higher than the contact rates estimate with beta-cyfluthrin and fluorescent powder. This difference in mosquito behaviour could be due to a change in the flow of human odours emanating from volunteer-occupied hut, which might be attenuated when tubes are screened with the inserts.

Overall, on the basis of its performance and residual activity, as well as commercial availability and existing regulatory approval in Côte d’Ivoire, beta-cyfluthrin was selected for the eave tube CRT. While having a pyrethroid insecticide in the eave tube might not seem an ideal option in an area of pyrethroid resistance, the resistance breaking properties of the electrostatic netting still enables use of a pyrethroid. Nonetheless, it will be important to monitor the potential for further selection for pyrethroid resistance. Moreover, screening for other active ingredients should be considered a priority to develop more sustainable resistance management strategies [24].

## Abbreviations

SET: Screening plus eave tubes
PVC: Polyvinyl chloride
CDC: Centers for Disease Control and Prevention
UV: Ultra-violet
BGLMs: Bayesian generalized linear models
GLMMs: Generalized linear mixed models
